# The Effect of Systemic Nitroglycerin Administration on the Kynurenine Pathway in the Rat

**DOI:** 10.3389/fneur.2017.00278

**Published:** 2017-06-14

**Authors:** Gábor Nagy-Grócz, Klaudia F. Laborc, Gábor Veres, Attila Bajtai, Zsuzsanna Bohár, Dénes Zádori, Annamária Fejes-Szabó, Eleonóra Spekker, László Vécsei, Árpád Párdutz

**Affiliations:** ^1^MTA-SZTE Neuroscience Research Group, University of Szeged, Szeged, Hungary; ^2^Faculty of Health Sciences and Social Studies, University of Szeged, Szeged, Hungary; ^3^Department of Neurology, Faculty of Medicine, Albert Szent-Györgyi Clinical Center, University of Szeged, Szeged, Hungary

**Keywords:** migraine, nitroglycerin, kynurenic acid, l-kynurenine, kynurenine pathway

## Abstract

The primary headache disorders include migraine, which is one of the most frequent neurological disorders, which influences more than 14% of the whole population. Despite the research efforts, its exact pathomechanism is not fully revealed, but evidence points to the role of glutamate and its receptors. Kynurenic acid is an endogenous glutamate receptor antagonist produced by the kynurenine pathway (KP). Tryptophan 2,3-dioxygenase (TDO) and indoleamine 2,3-dioxygenase (IDO) convert l-tryptophan to *N*-formyl-l-kynurenine, to be further transformed to l-kynurenine. Kynurenine aminotransferase-II (KAT-II), l-kynurenine hydrolase (KYNU), and l-kynurenine 3-monooxygenase (KMO) are key enzymes in the later steps of the KP. Nitroglycerin (NTG) administration serves as both human and animal model of migraine, causing the activation and sensitization in the trigeminal system. A previous study demonstrated a reduction of KAT-II expression following NTG administration in animals. The goal of current tests was to identify the potential modulatory effect of NTG on other metabolizing enzymes of the KP in the caudal trigeminal nucleus (TNC) of rats. Four hours following the intraperitoneal injection of NTG (10 mg/kg), the rats were perfused transcardially and the TNC was extracted for Western blotting. Western blot studies revealed that the expression of TDO2, IDO1, KYNU, and KMO decreased in the TNC. The results demonstrated that NTG is able to downregulate the KP, with a potential influence on the glutamatergic system as well, contributing to the development of trigeminal activation and sensitization in animals.

## Introduction

**Figure 1:**
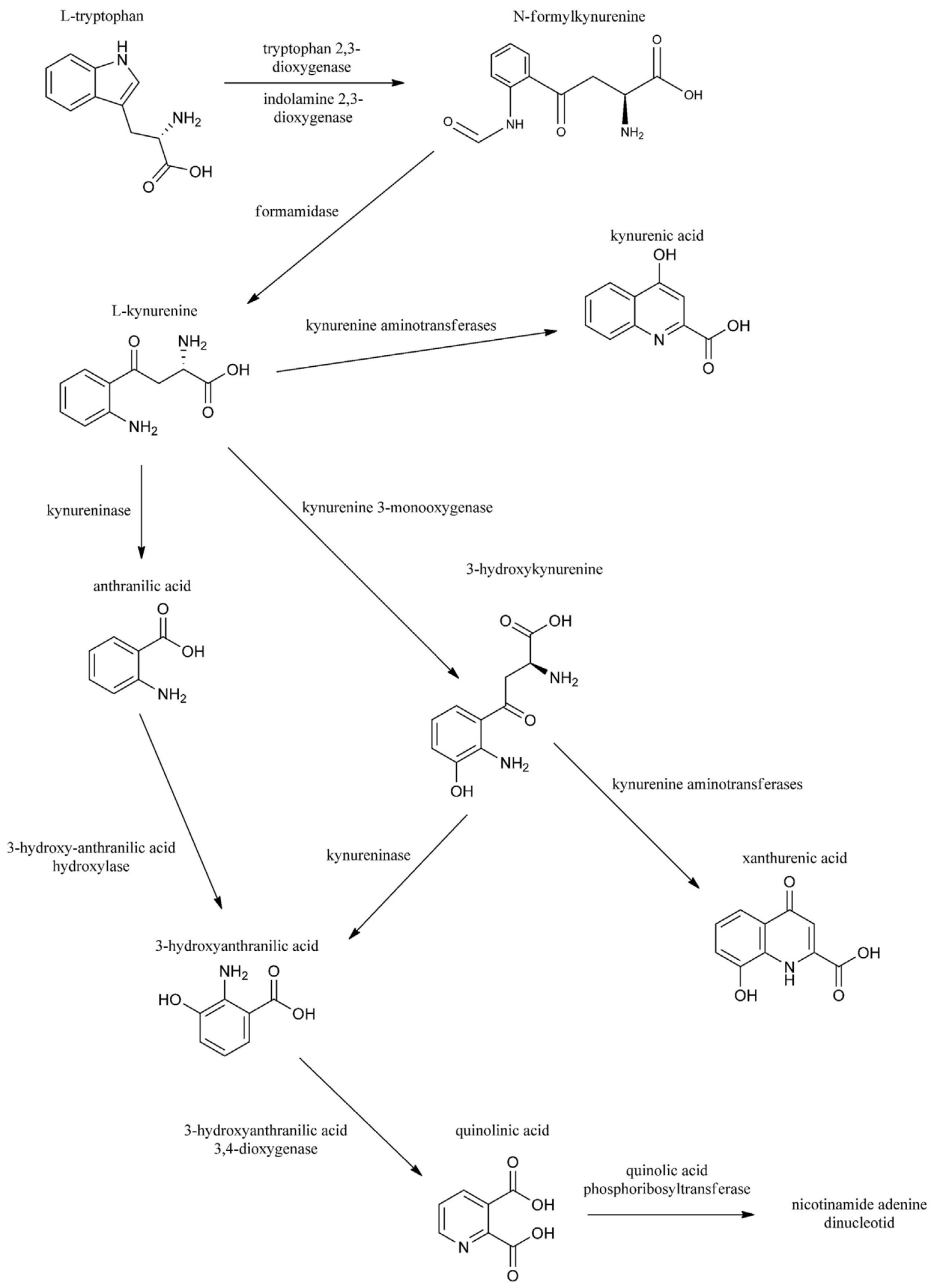
The kynurenine pathway.

Migraine is a common primary headache characterized by severe head pain and numerous concomitant symptoms, e.g., vomiting, nausea, photophobia, and phonophobia. The disease affects about 14% of the total population (1). The exact pathophysiology of the disorder is not fully understood, but it is well known that the activation and sensitization of the trigeminal system is essential during the attack (2). Several lines of evidence have been put forth to support the hypothesis that glutamate receptors, principally *N*-methyl-d-aspartate (NMDA) receptors, have a pivotal aspect in these phenomena (3). Kynurenic acid (KYNA) is a neuroprotective metabolite that interacts with glutamate receptors, aryl hydrocarbon receptor, and G protein-coupled receptor 35 and elicits anti-glutamatergic actions. Series of data confirm that KYNA and its analogs have anti-nociceptive effects in several migraine models (4–6), probably by attenuating the trigeminal activation and sensitization. The initial process in the kynurenine pathway (KP) is the transformation of tryptophan (Trp) to *N*-formyl-l-kynurenine by tryptophan 2,3-dioxygenase (TDO1, 2) and indoleamine 2,3-dioxygenase (IDO1,2): the rate-limiting enzymes of Trp metabolism. *N*-formyl-l-kynurenine is further converted by formamidase to l-kynurenine (l-KYN), which is converted to KYNA by kynurenine aminotransferases (KATs), to 3-hydroxykynurenine by l-kynurenine 3-monooxygenase (KMO), and to anthranilic acid by l-kynurenine hydrolase (KYNU). The other metabolite of the KP is quinolinic acid (QUIN). In contrast to KYNA, QUIN is an agonist of the NMDA receptors and can provoke neuronal death and also causes lipid peroxidation and generates reactive oxygen species (7, 8) (Figure 1).

Systemic administration of nitroglycerin (NTG) can be utilized as a human and animal model of migraine. NTG is enzymatically converted to nitric oxide (NO) in the body, probably by a mitochondrial aldehyde dehydrogenase (9). The administration of NTG is able to activate and sensitize the trigeminal system in humans and animals (10–12). In our previous study, it was demonstrated that NTG decreased the expression of kynurenine aminotransferase-II (KAT-II) (13), which converts l-KYN to KYNA in the brain thus reducing KYNA levels, contributing to the hyperactivity of NMDA receptors.

The goal of this study was to explore the issue of NTG on the expression levels of TDO2, IDO1, KYNU, and KMO enzymes in the caudal trigeminal nucleus (TNC).

## Materials and Methods

### Animals

We followed the directives for the Use of Animals in Research of the International Association for the Study of Pain and the policy of the European Economic Community (86/609/ECC). They were authorized by the local ethical committee of University of Szeged and the Scientific Ethics Committee for Animal Research of the Protection of Animals Advisory Board (XXIV./352/2012). 44 adult male Sprague-Dawley rats of 200–250 g bodyweight were used. The rodents were raised and maintained under standard laboratory conditions with tap water and regular rat chow available *ad libitum* on a 12 h dark–12 h light cycle.

### Drug Administration

The animals were separated into two groups (*n* = 5). The animals in the first group, called placebo group, received only the vehicle solution (physiological saline) as treatment. In the second group, the rats were treated with an intraperitoneal injection of NTG (10 mg/kg bodyweight, Pohl Boskamp).

### Western Blot Analysis

Four hours after the placebo/NTG injection, the animals were perfused transcardially with 100 mL phosphate-buffered saline and the dorsal horns of TNC segments (+1 and −5 mm from the obex) were extracted. The samples were stored at −80°C and they were sonicated in ice cold lysis buffer containing 50 mM Tris–HCl, 150 mM NaCl, 0.1% igepal, 0.1% cholic acid, 2 µg/mL leupeptin, 2 mM phenylmethylsulfonyl fluoride, 1 µg/mL pepstatin, 2 mM EDTA, and 0.1% sodium dodecyl sulfate. The homogenates were centrifuged for 10 min at 12,000 RPM at 4°C and supernatants were aliquoted and stored at −20°C until use. BCA Protein Assay Kit was used to measure protein concentration. Samples were mixed with sample buffer and were boiled for 3 min. Standard SDS polyacrylamide gel electrophoresis was performed with equal amounts of protein samples (20 μg/lane) loaded on 10% Tris–glycine gel and electrotransferred onto Amersham Hybond-ECL nitrocellulose membrane (0.45 µm pore size). Page Ruler Prestained Protein Ladder (10–170 kDa) was used to define approximate molecular weights. Non-specific binding was eliminated by blocking in Tris-buffered saline containing Tween 20 (TBST) and 5% non-fat dry milk for 1 h at room temperature. Then, membranes were incubated in TBST containing 1% non-fat dry milk and TDO antibody (LifeSpan BioSciences, LS-C111058, dilution: 1:500, incubation: overnight at room temperature), IDO antibody (Abcam, ab106134, dilution: 1:500, incubation: overnight at room temperature), KYNU antibody (Abcam, ab96365, dilution: 1:500, incubation: overnight at room temperature), KMO antibody (Abcam, ab83929, dilution: 1:4,000, incubation: overnight at room temperature), or glycerinaldehyde 3-phosphate dehydrogenase (GAPDH) antibody (Cell Signaling Technologies, 8884, dilution: 1:1,000, incubation: overnight at room temperature). On the following day, a horseradish peroxidase-conjugated anti-rabbit secondary antibody (Santa Cruz Biotechnology, sc-2030) in TBST containing 1% non-fat dry milk was applied for 2 h at room temperature. SuperSignal West Pico Chemiluminescent Substrate was used to visualize bands on Carestream Kodak BioMax Light film. Western blot protocol was developed based on previous experiments (4, 14–16).

An observer blinded to the experimental groups carried out the measurements. The detailed methods were described previously (13).

Films were quantified by Java ImageJ 1.47v analysis software (National Institutes of Health). The data were standardized to GAPDH.

### Statistical Analysis

Statistical analysis was carried out by SPSS Statistics software (Version 20.0 for Windows, SPSS Inc.). Normality was checked by Kolmogorov–Smirnov test, and group means were compared by independent *t*-test, with *p* < 0.05 taken as statistically significant. Group values are presented as means ± SEM.

## Results

### NTG Induced a Decrease in TDO2 Expression in the TNC

**Figure 2:**
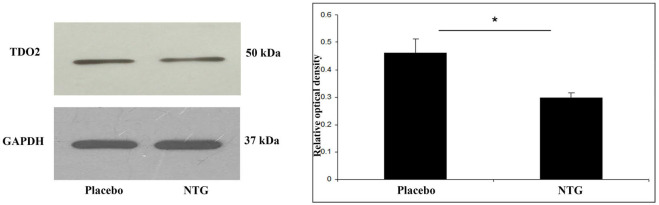
Western blot analysis of TDO2 and GAPDH protein from the TNC. The quantitative analysis shows that in the NTG group the relative optical density of TDO2 specific bands were significantly less pronounced compared with the placebo group. *p < 0.05; GAPDH, glyceraldehyde 3-phosphate dehydrogenase; NTG, nitroglycerin; TDO2, tryptophan 2,3-dioxygenase 2; TNC, caudal trigeminal nucleus.

TDO2 protein was identified at 50 kDa in Western blot assay. Densitometric analyses showed that the TDO2 bands were significantly decreased (*p* < 0.05) in the TNC after NTG administration as correlated with the placebo-treated animals (Figure 2).

### NTG Treatment Resulted in a Diminished IDO1 Expression

**Figure 3:**
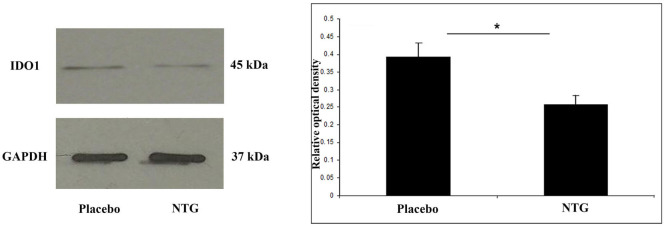
Western blot of IDO1 and GAPDH expression in the TNC. The quantitative analysis shows that in the NTG group, the relative optical density of IDO1-specific bands was significantly decreased compared with the placebo group. *p < 0.05; GAPDH, glyceraldehyde 3-phosphate dehydrogenase; IDO1, indoleamine 2,3-dioxygenase; NTG, nitroglycerin; TNC, caudal trigeminal nucleus.

A band characteristic of the IDO1 protein was referred at 45 kDa in Western blot assay. Densitometric analyses confirmed that the IDO1 bands were significantly weaker (*p* < 0.05) in the TNC after NTG administration as correlated with the placebo-treated animals (Figure 3).

### NTG Was Able to Reduce the Expression of KYNU

**Figure 4:**
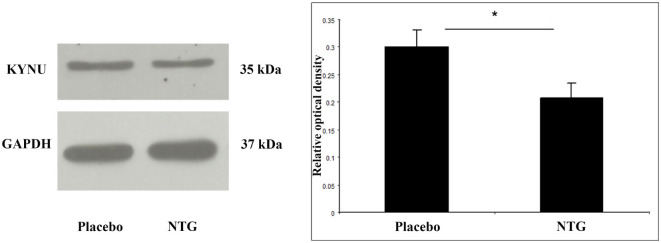
Representative Western blot bands and diagram of KYNU and GAPDH in the TNC. The quantitative analysis shows that in the NTG group, the relative optical density of KYNU-specific bands were significantly smaller compared with the placebo group. *p < 0.05; GAPDH, glyceraldehyde 3-phosphate dehydrogenase; KYNU, kynurenine hydrolase; NTG, nitroglycerin; TNC, caudal trigeminal nucleus.

We could determine a band at 35 kDa characteristic for the KYNU protein. In animals, which had received NTG, the density of KYNU protein bands was weaker in TNC segments (*p* < 0.05) as compared with the placebo-treated group (Figure 4).

### KMO Expression Was Lower after NTG Administration

**Figure 5:**
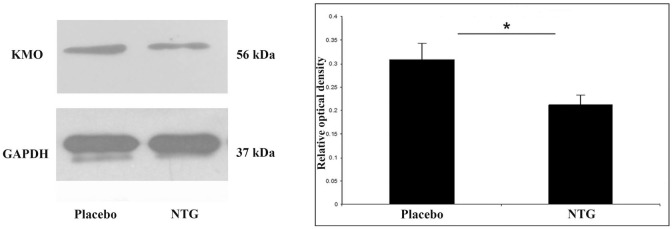
Illustrative Western blot bands and diagram of KMO and GAPDH in the TNC. The quantitative analysis shows that in the NTG group, the relative optical density of KMO specific bands was significantly weaker compared with the placebo group. *p < 0.05; GAPDH, glyceraldehyde 3-phosphate dehydrogenase; KMO, l-kynurenine 3-monooxygenase; NTG, nitroglycerin; TNC, caudal trigeminal nucleus.

l-Kynurenine 3-monooxygenase protein was identified at 56 kDa in Western blot assay. Densitometric analyses showed that the KMO bands were significantly decreased (*p* < 0.05) in segments TNC after NTG administration as correlated with the placebo-treated animals (Figure 5).

## Discussion

The current data demonstrated that NTG is able to decrease the expression of the KP enzymes in rat TNC. However, the question arises how NTG/NO can influence the KP. It is well known that the nitrergic system is able to alter the KP, e.g., NO can inhibit IDO expression *via* reversible binding to the active site in macrophages (17) and IDO activity is downregulated by NO production in bone marrow cells (18). The other hand, it is also known that the expression of KMO influences NO production in human HEK293 cells (19). Moreover, Backhaus and her colleagues showed in a mass spectrometry and NMR study that there is a direct interaction between kynurenine metabolites (e.g., 3-hydroxykynurenine and 3-hydroxyanthranilic acid) and NO (20).

It is also important to note that kynurenines have a crucial role in immune regulation (21). The transcriptional expression of IDO, KMO, and KYNU is also under the control of interferons (21), thus pro-inflammatory cytokines influence KP (22). Lögters and co-workers have shown that the ratio of kynurenine–tryptophan was increased in the blood of patients with posttraumatic sepsis (23), which pointed out that inflammation could modulate KP.

In this context, it is crucial that NO may cause neurogenic inflammation in the central nervous system. This is supported by observations that NTG was able to increase the expression of nuclear factor κB in the trigeminocervical complex of rats (13, 24), which is a key player in the inflammation process controlled by cytokines. In human studies, Tfelt-Hansen and his group demonstrated that infusion of NTG can trigger inflammatory response by inducing inflammatory mediators, which response was inhibited by the anti-inflammatory drug, prednisolone (25).

In the previous study, we demonstrated that NTG was able to decrease the expression of KAT-II, which produces KYNA. Our present and earlier Western blot data showed that NTG could reduce the expression levels of the KP enzymes.

Our findings are comparable with recent studies, which showed that the chronic migraine and cluster headaches are associated with altered levels of kynurenine metabolites, i.e., reduced levels of KYNA and l-KYN have been found in the serum of these patients (26, 27). These findings are in accordance with the theory of an increased release of glutamate probably yielding to a hyperactivity of glutamate receptors.

Migraine can be characterized by an increase in glutamatergic function (28), yielding fully activated NMDA receptors by the high glutamate levels, which might combine with small KYNA levels. Increased glutamate levels were found in the human cerebrospinal fluid, platelets, and plasma of migraine sufferers (29, 30).

To summarize the human and animal data, we can conclude that the KP is downregulated under the different types of headaches and thus possibly providing less KYNA. These data are in line with the theory of hyperactive NMDA receptors having a crucial role in the migraine pathophysiology. These receptors are key players in the mechanism of central sensitization (31), which have a pivotal role in the pathophysiology of migraine. Our present data strongly confirm that the KP has a relevant role in the pathomechanism of the trigeminal activation and sensitization, thus in the migraine pathology as well. In summary, influencing the KP provides a possible new target in the future therapy of migraine.

## Ethics Statement

We followed the directives for the Use of Animals in Research of the International Association for the Study of Pain and the policy of the European Economic Community (86/609/ECC). They were authorized by the local ethical committee of University of Szeged and the Scientific Ethics Committee for Animal Research of the Protection of Animals Advisory Board (XXIV./352/2012).

## Author Contributions

GN-G: participated in the design and implementation of experiments, statistical analysis, interpreted data, and wrote the manuscript. KL, GV, AB, ZB, DZ, AF-S, and ES: participated in the implementation of the experiments and statistical analysis. LV: participated in the design of the experiments and in the final approval of the version to be published. ÁP: participated in the conception and design of the experiments and in the interpretation of the data and writing. All authors: critical revision of the manuscript.

## Conflict of Interest Statement

The authors declare that the research was conducted in the absence of any commercial or financial relationships that could be construed as a potential conflict of interest.

## Abbreviations

GAPDH, glyceraldehyde 3-phosphate dehydrogenase; IDO, indoleamine 2,3-dioxygenase; KAT-II, kynurenine aminotransferase-II; KMO, l-kynurenine 3-monooxygenase; KYNA, kynurenic acid; KYNU, l-kynurenine hydrolase; KP, kynurenine pathway; l-KYN, l-kynurenine; NMDA, *N*-methyl-d-aspartate; NO, nitric oxide; NTG, nitroglycerin; PBS, phosphate-buffered saline; QUIN, quinolinic acid; TBST, Tris-buffered saline containing Tween 20; TDO, tryptophan 2,3-dioxygenase; TNC, caudal trigeminal nucleus; Trp, tryptophan.
