# Corrigendum: The Effect of Systemic Nitroglycerin Administration on the Kynurenine Pathway in the Rat

**DOI:** 10.3389/fneur.2020.01049

**Published:** 2020-09-24

**Authors:** Gábor Nagy-Grócz, Klaudia F. Laborc, Gábor Veres, Attila Bajtai, Zsuzsanna Bohár, Dénes Zádori, Annamária Fejes-Szabó, Eleonóra Spekker, László Vécsei, Árpád Párdutz

**Affiliations:** ^1^MTA-SZTE Neuroscience Research Group, University of Szeged, Szeged, Hungary; ^2^Faculty of Health Sciences and Social Studies, University of Szeged, Szeged, Hungary; ^3^Department of Neurology, Faculty of Medicine, Albert Szent-Györgyi Clinical Center, University of Szeged, Szeged, Hungary

**Keywords:** migraine, nitroglycerin, kynurenic acid, l-kynurenine, kynurenine pathway

In the original article, there was a mistake in Figures 2, 3, and 5 as published. **The authors became aware of the erroneous representation of the loading controls for the western blots of the figures. These errors were due to mistakes while generating the figures from templates of the western blot X-ray film images**. The corrected Figures 2, 3, and 5 appear below.

**Figure 2:**
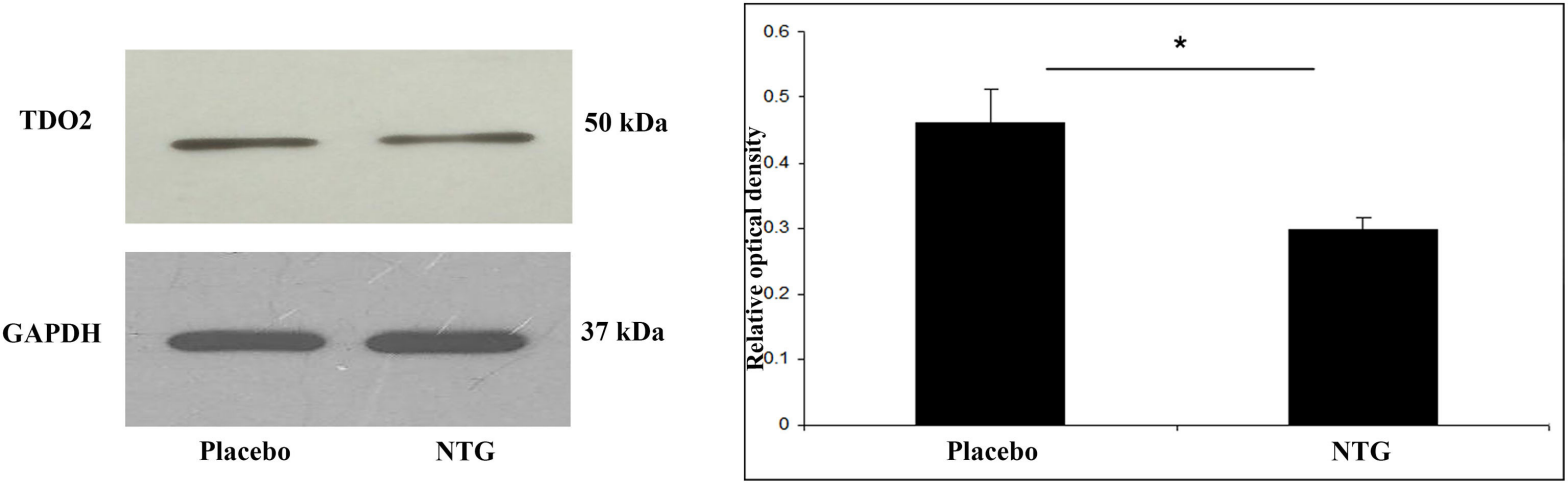
Western blot analysis of TDO2 and GAPDH protein from the TNC. The quantitative analysis shows that in the NTG group the relative optical density of TDO2 specific bands were significantly less pronounced compared with the placebo group. *p < 0.05; GAPDH, glyceraldehyde 3-phosphate dehydrogenase; NTG, nitroglycerin; TDO2, tryptophan 2,3-dioxygenase 2; TNC, caudal trigeminal nucleus.

**Figure 3:**
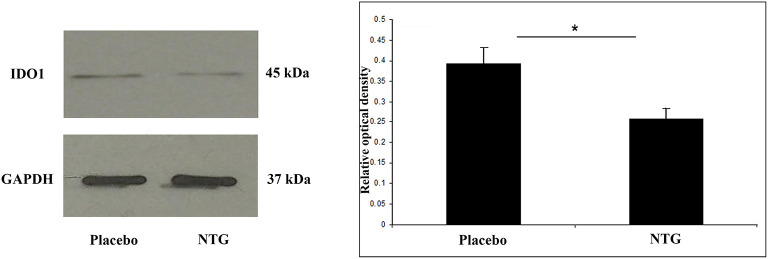
Western blot of IDO1 and GAPDH expression in the TNC. The quantitative analysis shows that in the NTG group, the relative optical density of IDO1-specific bands was significantly decreased compared with the placebo group. *p < 0.05; GAPDH, glyceraldehyde 3-phosphate dehydrogenase; IDO1, indoleamine 2,3-dioxygenase; NTG, nitroglycerin; TNC, caudal trigeminal nucleus.

**Figure 5:**
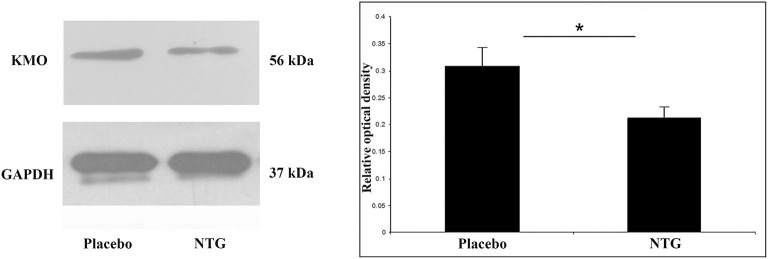
Illustrative Western blot bands and diagram of KMO and GAPDH in the TNC. The quantitative analysis shows that in the NTG group, the relative optical density of KMO specific bands was significantly weaker compared with the placebo group. *p < 0.05; GAPDH, glyceraldehyde 3-phosphate dehydrogenase; KMO, l-kynurenine 3-monooxygenase; NTG, nitroglycerin; TNC, caudal trigeminal nucleus.

The authors apologize for this error and state that this does not change the scientific conclusions of the article in any way.

